# Innovations of the ICD-11 in the Field of Autism Spectrum Disorder: A Psychological Approach

**DOI:** 10.32872/cpe.10005

**Published:** 2022-12-15

**Authors:** Kirstin Greaves-Lord, David Skuse, William Mandy

**Affiliations:** 1 Department of Psychology, Clinical Psychology and Experimental Psychopathology unit, University of Groningen, Groningen, The Netherlands; 2 Autism Team North-Netherlands, Jonx, Lentis Psychiatric Institute, Groningen, The Netherlands; 3 Great Ormond Street Institute of Child Health, University College London, London, United Kingdom; 4 Research Department of Clinical, Educational, & Health Psychology, University College London, London, United Kingdom; University of Zurich, Zurich, Switzerland

**Keywords:** Autism Spectrum Disorder, ICD-11, diagnostic process policies

## Abstract

**Background:**

This article aims to explain and elaborate upon the recently released ICD-11 criteria for Autism Spectrum Disorder (ASD, World Health Organization), which endorse a medical model.

**Method:**

We integrate insights from several disciplines (e.g., psychology, linguistics, sociology and lived experiences) to reflect the scientific and ethical insights derived from the biopsychosocial, neurodiversity perspective on autism.

**Results:**

First, we describe the core domains of ASD’s behavioural characteristics and then the lifetime, developmental perspective on the manifestations of these behaviours. Subsequently, we discuss potential underlying neuropsychology, related behaviours (i.e. associated features/conditions) and we consider some similarities and differences with the Diagnostic and Statistical Manual of Mental Disorders Fifth Edition (DSM 5, American Psychological Association).

**Conclusions:**

Recommendations for clinical application are provided. For instance, diagnostic classification in clinical practise should be a means to provide proper, suitable care, and therefore all diagnostic assessments should be used to tailor interventions and/or care to the capacities and genuine needs of the people that ask for professional help.

## Highlights

- Atypical responses to sensory stimuli are included as part of the diagnostic requirements in ICD-11, in contrast to ICD-10, where unusual sensory processing was not yet considered a core (diagnostic) feature.
- In ICD-11 it is recognized that some individuals with Autism Spectrum Disorder start to experience distress, impairment and overt social challenges once societal demands increase (e.g., during adolescence or adulthood).
- Unlike DSM-5, ICD-11 does not emphasize the criteria related to Disorders of Intellectual Development (ID; such as flipping objects, strong attachment or preoccupation with unusual objects, excessive smelling or touching of objects, echolalia, stimming).

## Current ICD-11 Definition, Criteria and Conceptualisations of Autism Spectrum Disorder

According to the current International system for the Classification of Diseases 11^th^ Revision (ICD-11) diagnostic requirements, in order to receive a classification of Autism Spectrum Disorder (ASD), a person’s behaviour should be characterised by three essential features. First, “persistent deficits in the ability to initiate and sustain reciprocal social interaction and social communication” (World Health Organization, 2019a). Second, by “a range of restricted, repetitive, and inflexible patterns of behaviour, interests or activities that are clearly atypical or excessive for the individual’s age and sociocultural context”. Atypical responses to sensory stimuli are now included in this domain, unlike ICD-10, where unusual sensory processing was not considered a core (diagnostic) feature. Third, “symptoms should result in significant impairment in personal, family, social, educational, occupational or other important areas of functioning” and, as in previous definitions, the onset should have been during early development. Yet, some individuals with ASD can function in many contexts through exceptional effort, such that their autistic characteristics are not apparent to others during childhood. ICD-11 recognises that overt symptoms are sometimes only fully manifest later, in adolescence or even adulthood, when social demands exceed capacities. Consequently, the condition can present clinically at all ages. ASD is a “lifelong condition, of which the manifestations and ­ impact are likely to vary according to age [developmental stage], intellectual ­ and language abilities, co-occurring conditions and environmental context”.

The ICD-11 is an international system for the Classification of Diseases. As such, it endorses a medical model, conceptualising Autism Spectrum *Disorder* as a medical condition with an inborn, for a substantial part, genetically inherited nature, while acknowledging that gene-environment interactions also play a pivotal role in neurodevelopment (classifying this category in the over-arching category of Neurodevelopmental Disorders). Although most people agree with this conceptualisation of neuro-biological aetiology, amongst a variety of stakeholders, the preference for a biopsychosocial model with more emphasis on how social factors affect functioning and wellbeing, is increasing (Bolis et al., 2017; Greaves-Lord et al., 2022). In such integrative accounts of ASD, an autistic person’s difficulties are not seen as simply caused by individual deficits; but rather are understood as arising from a poor fit between, on the one hand, the individual’s characteristics and, on the other hand, the demands placed on them by their environment (Mandy, 2022). According to this perspective, autistic symptoms are seen as a form of neurodiversity, and emphasis is placed on promoting functioning and wellbeing via environmental modifications that can improve person-environment fit.

In this article, we were invited to describe and reflect upon the recently released ICD-11 criteria, therefore, this will be the focus of the paper. Yet, in doing so, we will try to integrate insights from several disciplines (e.g., medical, psychological, linguistic, sociological and lived experiences), to reflect the scientific and ethical insights derived from the biopsychosocial, neurodiversity perspective on autism. We will first go into the core domains of ASD’s behavioural characteristics. Then we will emphasize the lifetime, developmental perspective on the manifestations of these core behaviours. Subsequently, we briefly discuss theories on the underlying neuropsychological mechanisms driving the core behaviours. Finally, we discuss related behaviours (i.e. associated features/conditions), consider similarities and differences with the Diagnostic and Statistical Manual of Mental Disorders Fifth Edition (DSM 5, American Psychiatric Association, 2013) and make some final remarks for clinical application.

## Social Communication

Individuals on the autism spectrum display the full range of intellectual functioning and language abilities; nowadays, especially in high-income countries, an ASD diagnostic classification is increasingly made in individuals who have normal-range verbal and non-verbal intellectual abilities (e.g., Lord et al., 2022; Zeidan et al., 2022).

The key features of an ASD comprise persistent deviations from the norms of social behaviour shown by most non-autistic people, including difficulties with initiating and sustaining social communication and reciprocal social interactions, and responding in a manner considered typical (conventional). Whilst there is a normal distribution of such abilities in the general population, people with an ASD are “outside the expected range of typical functioning”, when an individual’s age and level of intellectual development are considered (World Health Organization, 2019a). “Specific manifestations will vary according to the individual’s chronological age, verbal and intellectual ability”, and the overall profile of their autistic characteristics (World Health Organization, 2019a). There are, however, a number of key characteristics of interpersonal behaviour that are the essence of the condition. First and foremost, is the difficulty of spontaneously understanding the verbal or non-verbal social communications of other people, together with the tendency not to respond typically (conventionally) to those communications. It should be noted that autistic people and professionals are increasingly aware that many of the social difficulties ascribed to autistic people as simply reflecting their impairments, are better understood as reflecting the challenges of ‘cross-neurotype’ interactions (Chen et al., 2021). Autistic people may struggle to understand non-autistic people, but also, non-autistic people frequently struggle to empathise with autistic people. People with an ASD diagnosis vary in terms of their social motivation, although ICD-11 states that there is a tendency for them, compared to non-autistic people, to show less interest in social interactions, and be less likely to pay attention to other people’s verbal and non-verbal social cues. An important nuance to make here, is that although some autistic people show less involvement in social interaction, this might not necessarily be the result of lower social motivation, but rather it may be a consequence of exhaustion from trying to emulate a typical non-autistic style of interaction, known sometimes as camouflaging (e.g., Cook et al., 2021; Livingston et al., 2019). Moreover, there is a critical role of early communicative experiences in the development of individuals’ attention towards other people’s verbal and non-verbal social communication cues (Vernetti et al., 2018).

“Children vary widely in the age at which they first acquire spoken language and the pace at which their speech and language become firmly established” (World Health Organization, 2019a). Most children with early language delay eventually acquire similar language skills to their same-aged peers. Early language delay alone is not strongly indicative of ASD, unless there is also evidence of limited motivation to engage in social communication and of atypical social interaction skills (World Health Organization, 2019a).

An essential feature of ASD is persistent atypicality in how language is used and understood for social communication. People with an ASD typically do not follow non-autistic norms (conventions) in how they integrate their spoken language with complementary non-verbal cues, such as (considered) appropriate eye-contact, gestures, facial expressions, nodding in agreement, or other demonstrations of acknowledgement. Compared to non-autistic people, they are less likely to use body language to share a perspective, such as pointing to express interest in a distant object, or sharing attention in some external event or object. There is usually reduced tendency to initiate, join, or to sustain a conventional back-and-forth social conversation, which has its origins in early childhood. In general, people with an ASD have difficulty understanding and using language in social contexts that are dominated by non-autistic people, and are less likely to initiate and sustain reciprocal, purely social conversations (especially ‘chat’). The pragmatic language difficulties that are typical of ASD can manifest as misunderstandings of others’ language due to literal interpretations, together with speech that lacks ‘normal’ (i.e., non-autistic) prosody and emotional expressiveness, sometimes with a distinctly monotonous tone of voice, or contrastingly, with exaggerative expressiveness. Some autistic people are unaware that, to non-autistic people, their use of language sounds atypical, and may talk with such precision that it is considered pedantic, together with the use of an arcane vocabulary. In isolation, atypical language of this nature is only indicative; the diagnostic classification of an ASD requires there to be broad range of additional social reciprocity difficulties, as well as tendency towards inflexible behaviour and sensory sensitivities (see below).

In the context of social relationships with non-autistic people, especially with unfamiliar individuals, there can be limited social awareness, which can lead to behaviour that is not appropriately modulated according to the social context. Although people with ASD are often characterised as ‘lacking empathy’, the evidence for diminished empathic capacity in typical ASD is not strong. Some research shows altered affective empathy (e.g., Mazza et al., 2014), but, especially in cognitively able individuals, cognitive empathy can usually be present, although there may be an altered processing speed (i.e. due to a local rather than a global processing style, information is processed somewhat slower, but in more detail; Bölte et al., 2007). According to clinical observations of autistic adults, the empathic response may be over-developed (i.e., the tendency to experience high levels of emotional contagion). Moreover, whilst someone with ASD may not obviously be conventionally responsive to a non-autistic person’s feelings, autistic adults often explain their atypical reaction reflects a state of anxious confusion and/or indecision, rather than unawareness or disinterest.

Compared to non-autistic people, those with ASD are less likely to spontaneously share their interests with others, and may assume that others do spontaneously share their own interests and point of view (without the need to explicitly ask them). Given that in social life, non-autistic people are often highly intolerant of even small deviations from social norms, this can lead to challenges making and sustaining typical peer relationships. The impact of such peer problems changes from early childhood to adolescence. Intimate friendships with peers become more significant during adolescence, and difficulties building such relationships often become more overt at that time (e.g., Mandy, 2022). Isolation from or rejection by peers will usually have secondary consequences in terms of impaired mental health (e.g. social anxiety, depression or even trauma). Genuine pervasive lack of interest in making peer relationships is rare. Clinically, it is important to be aware that a young person’s withdrawal from social interactions may reflect social anxiety, and could be the result of persistent lack of acceptance by a peer majority non-autistic group. Furthermore, peer victimisation is a common experience for autistic people, and clinical assessment should always explore whether bullying is occurring, how it can be stopped, and its impact on the individual.

Also, non-autistic individuals “vary in the pace and extent to which they acquire and master skills of reciprocal social interaction and social communication” (World Health Organization, 2019a). A diagnosis of ASD should only be considered if there is marked and persistent difference from the expected range of abilities and behaviours in these domains given the individual’s age, level of intellectual functioning, and sociocultural context. Some individuals may exhibit limited/altered social interaction due to shyness (i.e., feelings of awkwardness or fear in new situations or with unfamiliar people, due to anxiety about negative social judgement), behavioural inhibition (i.e., being slow to approach or to ‘warm up’ to new people and situations) or behavioural disinhibition (i.e. impulsiveness). Limited social interactions in shy or behaviourally (dis)inhibited children, adolescents, or adults are not indicative of ASD. Shyness is differentiated from ASD by evidence of typical, non-autistic social communication behaviours in familiar situations (World Health Organization, 2019a).

## Repetitive, Stereotyped Behaviours and Sensory Interests

“Many children go through phases of repetitive play and highly focused interests as a part of typical development. Unless there is also evidence of impaired reciprocal social interaction and social communication, patterns of behaviour characterized by repetition, routine, or restricted interests are not by themselves indicative of Autism Spectrum Disorder” (World Health Organization, 2019a). Clinically significant evidence requires persistent “restricted, repetitive, and inflexible patterns of behaviour, interests, or activities that are clearly atypical and excessive for the individual’s age and sociocultural context” (World Health Organization, 2019a).

Typically, children with ASD are slower and/or less able to adapt to new experiences and circumstances. Strong reactions (often one of acute anxiety, distress and/or anger) can be evoked by changes to a familiar environment that, to non-autistic people, seem trivial, or in response to unanticipated events. Characteristic of the response to such unwelcome change and uncertainty is extreme discomfort which manifests in childhood as acute distress. This resistance to change also commonly manifests as the tendency to strongly adhere to particular routines. These may be geographic, such as the need to follow familiar routes, or may require precise timing, such as during mealtimes or when travelling. The tendency to engage in restricted and repetitive behaviours persists over time, although its frequency and overtness may diminish during adolescence. In contrast, insistence on ‘sameness’, can become more prominent in later life. Other aspects of this underlying need for consistency and predictability can be observed in terms of unusually strong adherence to rules (e.g., when playing games), as well as marked “and persistent ritualized patterns of behaviour (e.g., a preoccupation with lining up or sorting objects in a particular way” (World Health Organization, 2019a) or analysing/systemizing all sorts of information). Historically, such behaviours have been dismissed by non-autistic people as serving no apparent external purpose, but recent qualitative research with verbally fluent autistic individuals has revealed that the actions of organizing and systemizing can serve to regulate arousal. Thus, as their internal tension builds up, (e.g., in response to increasing social demands) an autistic person might start organizing or performing some systemic routine, in order to calm down (Greaves-Lord et al., 2022).

Specific repetitive or stereotyped behaviours will differ according to the developmental stage of the individual, but the tendency is usually life-long. In contrast, “repetitive and stereotyped motor movements, such as whole-body movements (e.g., rocking), atypical gait (e.g., walking on tiptoes), unusual hand or finger movements and posturing” (World Health Organization, 2019a), are more likely to be observed during childhood and are seen in situations of distress and excitement (i.e. hyperaoursal, see below). Such behaviours can also persist into adulthood, especially in autistic people with a co-occurring Intellectual Disability (abbreviated: ID).

Many individuals with an ASD develop fascinations with specific topics, objects or activities. In ICD-11, these are characterised as persistent preoccupations “with one or more special interests, parts of objects, or specific types of stimuli (including media), or an unusually strong attachment to particular objects (excluding typical comforters)” (World Health Organization, 2019a). The range of special interests is wide, and they may change from time to time during development. A key feature of the intensity of the special interests that are typical of ASD, is their pervasiveness and the fact that they disrupt an individual’s ability to conform to conventional norms within a social setting, to some extent. For example, everyday life may be adversely influenced by the need to pursue those interests. In childhood, this could have a negative impact on the family, as could the intense attachment to favoured objects (e.g., because of the distress engendered by their being left behind or lost). Nevertheless, it is important to recognise that these fascinations often enrich autistic peoples’ lives, with positive effects on identity and mood. Furthermore, such fascinations can engender skill and expertise that is valued in wider society.

The most recent addition to the diagnostic rubric of ASD symptoms (i.e., a change from ICD 10 to ICD 11) is the presence of lifelong strong and persistent hypersensitivity and/or hyposensitivity to sensory stimuli. Sensory sensitivities can include unusual interests in certain sensory stimuli, which may include sounds, light, textures (especially clothing and food), odours and tastes. Although a strong interest in spinning objects is often illustrated in assessment tool as characteristic of ASD, this clear exemplar of autistic behaviour is mainly observed in individuals with ID and delayed social-emotional development. A positive interest in sensory stimuli is less common than negative reactions to such stimuli, but a strong negative reaction to everyday sensory stimuli can be upsetting for the autistic person and also disruptive of family life. These typically include sensitivities to sounds, especially white noise such as hand dryers or vacuum cleaners. The sounds may not be especially loud; these reactions are most frequently observed in childhood. Other negative reactions can be observed to bright lights, certain clothing textures including labels, and especially food textures. Negative reactions to textures in food typically include the avoidance of mixed textures, requiring strict food separation. Although such behaviours are not exclusively observed in ASD, their severity and persistence, together with the consequent impact on everyday life, are more typical of ASD.

## Life-Course Perspective and Advice on Assessment

When individuals with suspected ASD present in adolescence or in adulthood, it is essential to perform an interview on developmental history, and not to rely exclusively on self-report or observations of current behaviour, however well-structured the observation. This is because one prerequisite for the diagnostic classification (although deliberately formulated in a nuanced way) is evidence that the onset of the atypical behaviours occurred during the early developmental period, typically toddlerhood/childhood (i.e., pre-school/primary school).

In contrast to ICD-10, in ICD-11 there is no longer the requirement of history of delayed onset of language, or clear evidence of autistic symptoms before/around the age of four to five years. This change reflects in part the fact that Asperger syndrome has been discontinued as a valid diagnosis; typically, individuals with normal-range verbal intelligence do not have delayed onset of language and they have been subsumed into the ASD diagnostic rubric. Also, it is now recognized that some individuals with ASD start to experience distress, impairment and overt social challenges once societal demands increase (during adolescence or adulthood).

Late onset symptoms of ASD and their differential diagnosis from personality disorders in adulthood are still a complex and controversial issue. Difficulties in inter-personal functioning (i.e., with understanding others’ perspectives, intimacy and self-regulation) are also characteristic of personality disorders. As we do not conventionally diagnose personality disorder in childhood, clear history of early (preschool) social communication difficulties, could be a differentiating feature. Enquiries should attempt to define exactly when the atypical social behaviours started to occur, but more importantly, under what circumstances. Early signs and predictors of later manifest ASD, such as a lack of/altered attention to eyes (Jones & Klin, 2013) and limited facial recognition (Eussen et al., 2015)/limited use of facial expressions, should be investigated.

At the time our conventional diagnostic instruments were developed, most clinically recognised children with autism were also experiencing generalized developmental delay (i.e., ID). Plateauing of social communication and language skills and lack of progress in their development characterises many such children. Yet the minority had a period of normal development (sometimes including age-typical language skills), but then lost their previously acquired skills, often in the second year of life. Such regression can be rapid, over a period of days or weeks, and usually leads to impaired language and social responsiveness. “Loss of previously acquired skills is rarely (spontaneously) observed after 3 years of age” (World Health Organization, 2019a), but can occur in acquired conditions such as encephalitis. If it occurs after age 3, it is more likely to involve a more generalized loss of cognitive and adaptive skills (including the loss of bowel and bladder control, and impaired sleep), as well as regression of language and social abilities (World Health Organization, 2019a). In rare cases of spontaneous regression, recovery takes place. This is usually slow (over months or years), and usually requires intensive interdisciplinary care that focusses on restoring the lost skills, including support for the development of speech/conversational, adaptive and regulatory skills. Asking and clarifying concrete examples of atypical development is therefore key when performing an interview on developmental history, and especially challenging when done only once the individual and caregivers involved are already older. Therefore, training such interviewing skills is essential when educating mental health professionals.

In preschool children, indicators of an ASD “often include avoidance of mutual eye contact, resistance to (conventional expressions of) physical affection, lack of social imaginary play, language that is delayed in onset, or is precocious” (World Health Organization, 2019a), but not used for conventional back-and-forth social conversation; social withdrawal, marked fascinations with topics that are sometimes notably unusual, and lack of age-typical social interaction with non-autistic peers, characterized by parallel play or apparent disinterest. “Sensory sensitivities to everyday sounds, or to foods, may overshadow the underlying social communication deficits” (World Health Organization, 2019a). These social characteristics are often first reported by a nursery or other preschool placement where the child’s behaviour is observed to differ significantly from the majority. Therefore, obtaining information from such sources (e.g., reports from infant care agencies/pre-school) can be of important additional value when charting the developmental history, especially in older cases.

In children with ASD without a Disorder of Intellectual Development (or general developmental delay), "social adjustment difficulties outside the home may not be detected until school entry or adolescence", when atypical social communication all-too-commonly leads to peer rejection, bullying and social isolation (World Health Organization, 2019a). "Resistance to engage in unfamiliar experiences and marked reactions to even minor change in routines is typical" (World Health Organization, 2019a). Furthermore, a strikingly strong "focus on detail as well as rigidity of behaviour and thinking" may be present. Secondary mental health problems are common, and symptoms of anxiety (i.e. social/specific phobia; e.g. Verheij et al., 2015) may become evident at this stage of development (World Health Organization, 2019a).

By adolescence, the capacity to cope with increasing social complexity in peer relationships at a period of ever-more demanding academic expectations is often overwhelmed. In some autistic individuals, their underlying social communication difficulties may be overshadowed by the symptoms of co-occurring mental and behavioural disorders. Depressive or anxiety symptoms are often a presenting feature (World Health Organization, 2019a), and restrictive eating disorders (including anorexia nervosa) become increasingly common in autistic girls at this age. Thus, clinicians should be aware of potential underlying ASD when performing diagnostic assessment in mental health settings.

In adulthood, the capacity for those with ASD to cope with complex and fluid cross-neurotype “social relationships can become increasingly challenged, and clinical presentation may occur when social demands overwhelm the capacity to compensate. Presenting problems in adulthood may represent reactions to (victimisation and) social isolation” (World Health Organization, 2019a). Also, they may reflect the challenges of planning and organising one’s professional and personal life, and regulating emotions, with less support than was received in childhood and adolescence. Compensation strategies may be sufficient to sustain dyadic relationships, but usually come under excessive strain in more complex group situations. “Special interests, and focused attention, may benefit some individuals in education and employment. Work environments may have to be tailored to the capacities (and sensitivities) of the individual. A first diagnosis in adulthood may be precipitated by a breakdown in domestic or work relationships” (World Health Organization, 2019a). As mentioned, if the individual is autistic, there is always history of at least some atypical signs in early childhood social communication and relationships, although this may only become apparent, or interpreted as such, in retrospect.

Because it is now recognised that ASD represents a more intense manifestation of the wide range of behaviours that are observed in the general population, it is critical to consider the impact of those symptoms on everyday life, before making a diagnosis. Diagnostic criteria, as outlined above, stipulate that autistic characteristics should “result in significant impairment in personal, family, social, educational, occupational or other important areas of functioning” (World Health Organization, 2019a; e.g., emotional/physical wellbeing). Some individuals with ASD can function well in many contexts, often through exceptional effort on their part, such that their autistic characteristics are ‘camouflaged’ and are not apparent to others. A diagnosis of ASD is still appropriate in such cases, especially when such exceptional effort is no longer achievable due to aging or changing social circumstances, during which the autistic characteristics might become more apparent to others over time. Camouflaging is commonly described by autistic people as exhausting and is associated with elevated risk for anxiety, depression and suicidality (Cook et al., 2021).

## Hypothesised Neuropsychological Mechanisms Driving the Core Behaviours Defining ASD

Although ASD is defined based on behavioural features, several theories exist on the neuropsychological mechanisms hypothetically underlying these behaviours. Classically, three main theoretical frameworks explaining underlying neuropsychological functioning were presented; Theory of Mind (ToM; e.g., Andreou & Skrimpa, 2020), Executive Functioning (EF; e.g., Demetriou et al., 2019) and Central Coherence (CC; e.g., López et al., 2008). Over time, nuances were made on how these theories each explain particular behavioural aspects of autism (e.g., Happé et al., 2006). More recently, theories have been proposed that combine, integrate and extend these theories, e.g. the Predictive Coding account (PC; e.g., Van de Cruys et al., 2014) and the Polyvagal Theory (PT; e.g., Brown, 2020). Given the scope of this article, we cannot go into detail on all these accounts, nor can we mention the abundant literature. However, we will briefly explain these theories and illustrate them with examples of behaviours seen in autistic people, so that clinical psychologists can a) better understand what mechanisms might be driving certain behaviours, and b) use this to increase the understanding of autistic people they support.

Firstly, ToM refers to the ability to formulate hypotheses on how other people feel, think and thus behave; i.e. mentalizing. Autistic people might sometimes respond differently than conventionally would be expected. Such responses can however be better understood, when being aware that - depending on the circumstances - the response might be either mostly to the verbal information that was primarily processed, or to the visual information that was mainly processed (e.g., Chung et al., 2014).

Secondly, EF refers to a set of capacities used to *consciously* plan ahead, meet goals, display self-control, etc. Speculatively, more *unconscious*, automatically driven cognitive distortions might appear in case of cognitive overload in autistic people (e.g., Autistica, 2021). Sometimes, autistic people show the tendency to categorize things or people as all good or all bad, all right or all wrong (sometimes referred to as 'dichotomous ­ thinking'), rather than - at that instance - being able to *consciously* notice the possibilities in between, sometimes referred to dichotomous thinking.

Weak CC refers to difficulties in ‘seeing the bigger picture’, but rather an associative, non-linear thinking style in autistic people (e.g., Grandin, 2009). Simply put, some people might mainly have a global (bigger picture) processing style, while other (autistic) people might mainly have a local (detail-focussed) processing style (Bölte et al., 2007).

The idea of CC was taken further in PC theory. This theory of brain function stipulates that the brain is constantly generating and updating a mental model of the environment (e.g., Pellicano & Burr, 2012). This model is used to generate predictions of sensory input that are compared to actual sensory input. This comparison results in prediction errors that are then used to update and revise the mental model. An autistic person might be focussed more on the actual sensory input and their brain might be constantly working to minimize the gap between the prediction and actual sensory input. As such, this theory might explain why some autistic people have more intolerance of uncertainty, given the larger prediction errors and the cognitive resources it takes to try and solve these. Finally, although the PT (Porges, 1995) is not yet well substantiated empirically, it’s popularity is growing amongst some clinical practitioners and autistic people, as it is relatable. Therefore, we discuss it briefly. Polyvagal theory takes its name from the vagus, a cranial nerve that is the primary component of the parasympathetic nervous system. The autonomic nervous system (ANS) has two parts; the sympathetic nervous system, which is mostly activating (“fight or flight”), and the parasympathetic nervous system, which exists of two distinct branches: a "ventral vagal system" which supports social engagement, and a "dorsal vagal system" which supports immobilisation behaviours, both “rest and digest” and defensive immobilisation or “shutdown”. Behavioural responses that derive from the hybrid state of activation and calming are key to the ability to adaptively socially engage. It is speculated that in autistic people, the ANS might (at times) be dysregulated, which could explain emotional melt downs or shut downs in autistic people. Again, we emphasize that in this section we did not provide an extensive explanation of all neuropsychological concepts. Rather, we illustrated some behaviours seen in autistic people and tried to stimulate readers to think about their assumed neurobiological origins. In clinical practice, for most autistic people it is key to connect abstract, neuropsychological concepts to very concrete day-to-day personal experiences, to ‘digest’ these explanations fully (e.g., Gordon et al., 2015). Thus, in psycho-education, it is essential to help autistic people make these translational connections.

## Further Features and Disorders

Some individuals with an ASD experience delay in the development of their intellectual abilities, and qualify for a diagnosis of ID. In countries with well-established facilities for the assessment of autistic symptoms, and with experience in the manifestations of the condition among individuals with good verbal skills, individuals with ID are a minority of those diagnosed with ASD. By contrast, in more under-served areas, those with ID constitute the majority people diagnosed with ASD. “If present, a separate diagnosis of Disorder of Intellectual Development should be assigned, using the appropriate category to designate severity (i.e., Mild, Moderate, Severe, Profound, Provisional). Because social difficulties are a core feature of Autism Spectrum Disorder, the assessment of adaptive behaviour as a part of the diagnosis of a co-occurring Disorder of Intellectual Development should place greater emphasis on the intellectual, conceptual, and practical domains of adaptive functioning than on social skills” (World Health Organization, 2019a). Self-injurious behaviours (e.g., hitting one’s face, head banging) occur more often in autistic people with co-occurring Disorder of Intellectual Development, perhaps because they represent attempts to express and communicate painful feelings, in the absence of verbal means.

Even among individuals with normal-range intellectual abilities, profiles of specific cognitive skills in ASD as measured by standardized assessments, may show striking and unusual patterns of strengths and weaknesses that are highly variable from individual to individual. Clinical experience teaches that such a ‘spikey profile’ of cognitive strengths and difficulties can affect learning and adaptive functioning to greater extent than would be predicted from the overall scores on measures of verbal and non-verbal intelligence, yet more research on this matter is needed to substantiate such clinical claims. Isolated difficulties in intellectual functioning that are associated with ASD include slow/different processing speed/style (Bölte et al., 2007) and limited verbal or non-verbal working memory, which may occur in the presence of strong verbal and/or visuospatial skills in other domains.

"The degree of impairment in functional language (spoken or signed) should be designated with a second qualifier. Functional language refers to the capacity of the individual to use language for instrumental purposes (e.g., to express personal needs and desires). This qualifier is intended to reflect primarily the verbal and non-verbal expressive language [difficulties] present in some individuals with Autism Spectrum Disorder” (World Health Organization, 2019a), and not the atypical pragmatic language that is a core feature of the condition. ICD-11 requires the assessment of whether the individual has a degree of functional language impairment (spoken or signed) relative to their age in the following terms: i) with mild or no impairment of functional language; ii) with impaired functional language (i.e., not able to use more than single words or simple phrases); iii) with complete, or almost complete, absence of functional language (World Health Organization, 2019a).

It is important to note that the observable manifestation of ASD will be different at different developmental stages (as discussed above), as well as in different groups (e.g., males versus females versus gender-diverse individuals, or those with and without ID). For instance, parental or caregiver concerns about intellectual or other developmental delays (e.g., problems in language and motor coordination) often characterise the presentation in young children during the preschool period. When there is no significant impairment of intellectual functioning, the presentation to clinical services is often prompted by staff at nursery school, who have observed unusual social or other behaviour. In middle childhood, there may be prominent symptoms of anxiety, including social anxiety disorder, school refusal, and specific phobia (Verheij et al., 2015). During adolescence and adulthood, depressive disorders are a common presenting feature. For women, a restrictive eating disorder can drive engagement with mental health services, with their underlying ASD and/or associated social trauma only being identified later (Bentz et al., 2022). Across all ages, there is strong co-occurrence with attention deficit/hyperactivity disorder, and in males impulsive and disruptive behaviour often prompt referral (especially in middle childhood), although in females the symptoms are more likely to be related to attention difficulties, rather than impulsivity or hyperactivity. Consequently, it is important to be aware that ASD commonly co-occurs with other mental, behavioural or neurodevelopmental disorders across the lifespan. In a substantial proportion of cases, particularly in adolescence and adulthood, it is the co-occurring disorder that first brings the autistic individual to clinical attention. Some people with ASD are capable of functioning even in environments that are poorly adapted to accommodate them, by making an exceptional effort to compensate for their symptoms during childhood, adolescence or adulthood (i.e., ‘camouflaging’). Such camouflaging requires sustained effort, is more typical of females (although it is common in all genders), and can have deleterious impact on mental health and well-being (Cook et al., 2021).

“Some young individuals with Autism Spectrum Disorder, especially those with a co-occurring Disorder of Intellectual Development, develop epilepsy or seizures during early childhood with a second increase in prevalence during adolescence. Catatonic states have also been described. A number of medical disorders such as Tuberous Sclerosis, chromosomal abnormalities including Fragile X Syndrome, Cerebral Palsy, early onset epileptic encephalopathies, and Neurofibromatosis” are associated with an ASD diagnosis (World Health Organization, 2019a), with or without a co-occurring Disorder of Intellectual Development. Genomic deletions, duplications and other genetic abnormalities are increasingly described in individuals with ASD, some of which may be important for genetic counselling. Prenatal exposure to valproate is also associated with an increased risk of ASD (World Health Organization, 2019a).

Recently, there is growing recognition of the fact that people with ASD more frequently develop more severe physical illnesses, in the worst case resulting in relatively early death, as compared to other people from the general population. Potentially, this might reflect the fact that autistic people experience high levels of stress, due to having to live in environments that are poorly designed to accommodate them, with consequent elevated levels of mental health, suicidality and substance use problems. Poor physical health outcomes could reflect a combination of two underlying causes. First, autistic people might have a limited capacity to sense and ­recognize early physical symptoms. This might be due to limited interoception, i.e. hypo-sensitivity or­ a limited inclination to direct their attention towards internal stimuli of the body (e.g. Garfinkel et ­ al., 2016). Secondly, they might be reluctant to communicate any concerns they have about their physical health to professionals. This might result in their initially not seeking access to medical services, as well as limiting their action in following up any subsequent referral to medical specialists. Research on this topic is still ongoing. Nevertheless, it is important that mental health professionals are aware that there is potentially limited somatic awareness in autistic clients. They should therefore pro-actively bring up the topic of their client’s physical health. Psychologists should consider referral to a medical specialist when an autistic client complains about somatic symptoms, and should be aware of their potential professional biases. Faced with an autistic client who has somatic symptoms they should not automatically assume a psychological explanation, but be aware that an alternative physical­ condition could be present, and that condition should be adequately investigated. The prevalence of premature mortality affecting people on the autism spectrum, which is excessive, could be attributable at least in part from these risk factors. 

## Comparison Between ICD-11 and DSM-5

Both systems of diagnosis differ substantially from previous versions (ICD-10 and DSM-IV and DSM IV TR). There are differences in their conceptualization of ASD as a broad category comprising many different conditions (not yet identified, the 'autisms'), and in terms of specific phenotype requirements. Hence the agreed term ASD, reflecting the heterogeneity of those conditions. Both systems recognize that ASD is a set of symptoms that exist on a continuum that blends into normal variation, and they also consider the fact that at one extreme end there is a subset of conditions that are associated with identifiable biological substrates (largely genetic, but also some environmentally induced risks). The greatest difference between the ICD-11 and DSM 5 diagnostic systems is not in the social communication aspects of the condition, but in the patterns of restrictive, repetitive, and inflexible patterns of behaviour that are regarded as atypical.

The blurry boundaries between ID and ASD bedevils research. Experts who are looking at genetic risk factors continue to have a heated debate about whether certain genetic anomalies increase risk for ASD or ID or both. ICD-11 criteria are cognizant of the fact that nowadays most diagnoses of ASD are made in individuals who are of normal-range intelligence. Accordingly, B-scale symptoms are defined in a way that reflects behaviours that are seen in those individuals (more broadly ranging than is discussed in DSM-5). Unlike DSM-5, ICD-11 does not emphasize the ID-related criteria (such as flipping objects, strong attachment or preoccupation with unusual objects, excessive smelling or touching of objects, echolalia, stimming; WHO, 2019b). The associated limited enquiry about symptoms of Repetitive, Restricted and Stereotyped behaviour (RRSB) is one of the reasons why there was, under the former DSM-IV TR criteria, such high prevalence of Pervasive Developmental Disorder – Not Otherwise Specified ('PDD-NOS'). By broadening the criteria and introducing concepts such as 'Lack of adaptability to new experiences and circumstances...' ICD-11 has aimed to reduce the perceived lack of sensitivity of the DSM-5 criteria to cognitively able and older individuals.

Intellectual disability is conceptualized as a homogeneous condition in DSM-5. It is said that ASD may be difficult to differentiate from ID in very young children (under the heading Differential Diagnosis), but this statement exemplifies the problem that in the USA the terms are much closer aligned than the developers of ICD-11 considered to be appropriate. DSM-5 does not make distinctions between levels of intellectual impairment. In ICD-11, as discussed, there is the possibility to record an associated Disorder of Intellectual Development, and this should be assigned a degree of severity.

DSM-5 criteria state that, to make an ASD diagnosis, the atypical social communication should be more marked than would be anticipated from the individual's developmental level when any associated ID is considered. In ICD-11 a similar statement is made. Both diagnostic systems acknowledge that it is important to distinguish the lack of adaptive behaviours that are indicative of generalized learning disabilities from the specific difficulties that are experienced by individuals with ASD. The difference in emphasis between the systems reflects the expectation in the US that it is important to identify ASD symptomatology in those with ID, whereas in ICD-11 the emphasis is on the importance of identifying intellectual impairment in those with a primary diagnosis of ASD.

In DSM-5 a differential diagnosis is made between ASD and Social (Pragmatic) Communication Disorder, a condition that does not exist in ICD-11. The developers of ICD-11 criteria were not convinced that a specific disorder of this nature could be differentiated clearly from atypical social communication that is associated with ASD, nor from varieties of Specific Language Impairment (Mandy et al., 2017). ICD-11 records the degree of impairment of functional language at three levels, but this distinction is not treated as a differential diagnosis. That decision, to record three levels of impairment appears to be similar, but more structured, than the DSM-5 stipulation to use the specifier 'with or without accompanying language impairment' with an injunction to assess the current level of language and describe it. The choice of three levels reflected the need to be more explicit for clinical purposes, and the ICD-11 developer’s estimate that this distinction could be made reliably.

Both systems of diagnosis require the recording of loss of skills. In ICD-11 there is a qualifier that records whether there is loss of previously acquired skills, or not. DSM-5 discusses loss of skills in the context of Development and Course and distinguishes social from loss of other skills (such as toileting or motor skills). ICD-11 acknowledges that the pattern of skill loss will be different at different stages of development.

DSM-5 has a section on differential diagnosis which implies that it is possible that ASD could be confused with other diagnoses, such as selective mutism or ADHD. ICD-11 has taken a different approach, recognizing that these conditions can (and frequently do) co-occur. Hence, in ICD-11 they are included in a section that uses the term 'Boundaries with Other Disorders and Conditions’. The guidelines in ICD-11 provide greater detail than DSM-5 about the distinction between conditions that may present with an autism-like phenotype.

## Towards Intervention to Improve Quality of Life and Functioning

In our view, the diagnostic classification of ASD should always inform and serve proper, suitable interventions and support aimed at improving the wellbeing and functioning of the autistic person. Thus, clinical psychologists should remain aware that diagnostic classification is not a purpose in itself. Therefore, as part of the diagnostic assessment process, clinicians should perform assessments with a purpose in mind. If the goal is to primarily acquire new insights for scientific/applied research and/or related mental health care innovations, that purpose of potential additional assessments should be transparently communicated to all involved. Diagnostic classification in clinical practise should be a means to provide proper, suitable care, and therefore all diagnostic assessments should be used to tailor the interventions and/or care to the capacities and genuine needs of the people that ask for professional help. Even though ASD is conceptualized as predominantly inborn, so genetically determined condition, the interaction with social factors is more and more recognized both in society as well as in research. As such, interventions to help autistic people should not simply focus on effecting change in the individual, but should also include steps to improve person-environment fit by making adaptations to the environment. Furthermore, intervention targets should be identified collaboratively with the client and their family, and will often concern improving wellbeing, mental health and societal functioning. Whilst practice may need to be adapted to promote access and inclusion for autistic clients, mental health care providers are in a good position to use their clinical skills to offer effective help. There is growing evidence-base for psychological treatment procedures and social support interventions. Recommendations regarding suitable methods for treatment and support with sufficient evidence as well as preference base will be provided in a future follow up article.
